# A complex behaviour change intervention delivered by dental nurses: mixed-methods fidelity assessment of the RETURN intervention

**DOI:** 10.1186/s13063-025-08856-0

**Published:** 2025-05-13

**Authors:** V. Lowers, P. Christiansen, B. Young, J. Hennessy, R. V. Harris

**Affiliations:** 1 Department of Public Health, Policy and Systems, Institute of Population Health, University of Liverpool, Liverpool, L69 3GL UK; 2 Department of Psychology, Institute of Population Health, University of Liverpool, Liverpool, UK

**Keywords:** Intervention fidelity, Behaviour change, Pragmatic randomised controlled trial, Dental team

## Abstract

**Introduction:**

Behaviour change interventions delivered in dental settings could be useful in reducing oral health inequalities. Pragmatic randomised controlled trials testing interventions, however, are vulnerable to problems with internal and external validity. Intervention fidelity strategies are helpful to address methodological problems and to provide scientific assurances about trial results. This paper sets out the intervention fidelity assessment of the RETURN intervention which was delivered in dental settings to promote planned dental visits.

**Methods:**

The assessment was guided by the National Institutes of Health Behaviour Change Consortium intervention fidelity framework domains of training and delivery. A mixed-methods design was selected, using quantitative data collected during intervention delivery training, together with qualitative observations (*n* = 58), interviews (*n* = 13) and audio-recordings of intervention delivery sessions (*n* = 472). Data were analysed separately using descriptive statistics and multiple logistic regression for the quantitative data and reflexive thematic analysis for the qualitative data. Data were integrated to provide a comprehensive fidelity assessment.

**Results:**

Dental nurses were successfully trained to deliver the RETURN intervention; training was successfully standardised, and skills drift minimised. Training presented challenges, and not all nurses achieved competency sign-off to deliver the intervention independently. Nurse characteristics such as dental nursing experience, wider trial procedures and confidence were all found to impact training success. The RETURN intervention was judged to have been delivered with high levels of fidelity, despite few interventions reaching the pre-determined fidelity threshold. Fidelity levels of between 75% and 85% were achieved across intervention domains. Interventionist, intervention dose and intervention topic (dental visiting barrier) were all found to have a relationship with fidelity levels.

**Conclusions:**

Dental nurses can be trained to deliver a brief behaviour change intervention alongside their usual clinical roles, and this can be delivered with a high degree of intervention fidelity. The results from this fidelity assessment provide assurances about the scientific validity of the RETURN trial results. Recommendations about the suitability of dental nurse interventionists within future dental trials are discussed.

**Trial registration:**

ISRCTN 84666712. Registered on 12/04/2021.

**Supplementary Information:**

The online version contains supplementary material available at 10.1186/s13063-025-08856-0.

## Introduction

Dental diseases are amongst the most prevalent non-communicable diseases worldwide, even though they are preventable [1]. Studies consistently show that oral health-related behaviours such as diet, toothbrushing and regular dental visiting not only affect oral health, but also intersect with health inequalities [2–7]. Regular dental visits support the prevention and management of dental disease by allowing early preventive interventions (for example, applying fluoride to support remineralising early tooth decay and delivery of tailored advice to support individuals’ skills and behaviours such as their toothbrushing routines [8]). Unfortunately, lower socio-economic groups are the least likely to use dental services in a planned way, even though they are more likely to have the highest need [5].

The inteRventions to rEduce inequaliTies in the Update of Routine deNtal care (RETURN) was a pragmatic randomised controlled trial (pRCT) developed to test a brief behaviour change intervention (BCI) designed to support patients in visiting the dentist for planned care [9, 10]. It was delivered by dental nurses whose routine clinical responsibilities would primarily include providing chairside assistance to dentists, such as mixing materials and providing suction whilst the dentist is operating in the mouth. Some dental nurses in the United Kingdom (UK) also provide health education advice to patients independently [11] but this is not universal. Dental nurses in the UK typically have varied educational backgrounds depending on the training route undertaken, with training entry educational requirements relatively low [12]. This is an important consideration for research involvement and training.

Intervention fidelity should be considered in trials delivering BCIs to ensure scientific rigour by enhancing the reliability and validity of interventions under test [13, 14]. It is recognised good practice to include details about fidelity when reporting pRCTs [15, 16]. Intervention fidelity studies assess whether an intervention was delivered as intended, ensuring that observed study outcomes are attributable to the intervention itself rather than any other reasons (i.e. inconsistent delivery) [17]. This information is vital when considering which interventions should be translated into clinical practice [13].

Intervention fidelity also plays a critical role in treatment effects. A recent meta-epidemiological review of complex rehabilitation interventions tested in RCTs found that higher intervention fidelity was linked to smaller but more precise treatment effect estimates, and lower or absent fidelity reporting was associated with overestimated treatment effects. This highlights the importance of fidelity in ensuring that interventions adopted into practice are truly effective. The authors of that review concluded that caution should be exercised when interpreting the effectiveness of complex interventions where fidelity is either unmonitored or unreported [18].

Reviews of oral health interventions have consistently identified methodological limitations within the literature. They highlight the need for improved ‘quality control of interventions’ [19] and call for ‘higher-quality’ studies, particularly in terms of intervention delivery reporting [20]. Intervention fidelity strategies offer a viable approach to strengthening the evidence-base for complex oral health interventions, particularly in a challenging setting such as primary care dentistry.

A recent review exploring intervention fidelity in primary dental care found that reporting is poor and the use of theoretically derived fidelity frameworks is limited [21]. Where intervention fidelity strategies are absent, methodological issues can arise. For example, a trial evaluating the delivery of oral health risk messages to patients did not utilise any intervention fidelity strategies and the authors recommended placing greater emphasis on improving the quality of intervention delivery, as site effects were observed across different dental practices [22]. Therefore, intervention fidelity remains an under-reported and under-utilised methodological tool within complex BCI dental research, and further efforts are needed to methodologically improve the evidence-base for complex interventions in dental trials.

The National Institutes of Health Behaviour Change Consortium (BCC) framework provides a structured approach to intervention fidelity in BCIs. It recommends using strategies to enhance and monitor fidelity across five domains: design, training, delivery, receipt and enactment [13, 14]. The framework recommends the targeted use of strategies such as ‘ensure interventionist skill acquisition through the use of pre-defined training criteria and the provision of feedback and support’ or ‘ensure adherence to the intervention protocol through the use of audio-recorded intervention session monitoring’ [13].

The current study, which was embedded within the RETURN trial, reports the fidelity assessment of the RETURN intervention focusing on the BCC domains of training and delivery. The RETURN intervention fidelity strategy was developed using data from the RETURN feasibility study [23] and the BCC recommendations. The full strategy is published elsewhere [24].

This fidelity assessment aimed to (1) determine how well the RETURN training programme was implemented by exploring the BCC goals of training standardisation, skill acquisition and skills drift; (2) determine how closely the RETURN intervention was delivered as it was intended through a fidelity checklist and ‘dose’ monitoring; and (3) present fidelity assessment results to contextualise the forthcoming findings of the RETURN trial.

### The RETURN trial

The RETURN trial was a two-arm, parallel group, 1:1 randomised, individual level, pRCT delivered in 13 NHS general dental practices and a dental hospital urgent clinic in North-West England, UK. Trial participants were recruited opportunistically when seeking urgent dental care at one of the RETURN sites. Trial inclusion criteria were (1) aged 18 years + , (2) had not visited a dentist for routine care within the last 2 years, (3) able to provide contact details to facilitate follow-up, (4) adequate understanding of spoken and written English and (5) responsible for making their own dental appointments [10]. A total of 1179 participants were recruited between August 2021 and September 2022.

Dental nurses were responsible for delivering the intervention (which took approximately 15 min) and the conduct of all trial procedures. These included eligibility screening, consenting, randomisation and data collection which took around 30 min to complete using a combination of online and paper-based methods.

### The RETURN intervention

The RETURN intervention is described fully elsewhere [9, 24]. Briefly, the intervention comprised two elements:

1. A ‘patient pack’ made up of materials such as booklets and 1–2-min videos with embedded behaviour change messages. Intervention sessions culminated in a written goal setting and action planning exercise, following SMART principles [25]. A text message was sent to participants a week or two post-intervention, reiterating the goal and action plan.
2. A structured behaviour change conversation facilitated by trained dental nurses, guided by motivational interviewing (MI) spirit [26]. Participants were offered a choice of six common barriers to routine dental visiting to select from (cost, lack of time, not thinking to go to the dentist when not in pain, embarrassment, lack of trust in dentists and dental anxiety), with one serving as the focus for the session.

### RETURN sites

RETURN sites were informed of the study through local dental networks and expressions of interest invited. The sole inclusion criterion was that sites were those providing urgent dental appointments to new patients. Interested practices attended an online information evening outlining the study. Dental nurses as well as practice owners were encouraged to participate to foster dental nurse engagement upfront. All sites expressing interest were included, although one withdrew before the study commenced without providing a reason. Of the 14 participating sites, 57% [8] were in the 10% most deprived areas of the UK [27]. Sites were predominately urban, offering between 3 and 30 urgent dental appointment slots per day. Most sites provided in-hours urgent dental care, with one providing out-of-hours care.

### Dental nurse interventionists

Twenty-five dental nurses and receptionists with prior nursing experience were recruited across participating sites to deliver the RETURN trial alongside their clinical responsibilities. The research team had no role in selecting the participating nurses. Instead, these decisions were determined by site-specific practical constraints, such as staff availability. Additionally, three research dental nurses provided supplementary ‘float’ recruitment support.

### The RETURN training programme

Training was tailored to the primary dental care setting, considering both clinical time constraints and research delivery experience levels of the dental teams [28, 29]. Trainees attended training elements that directly related to their allocated research roles. See Additional file 1 for a full training description detailing the different training components and the required attendees.

### Phase 1: intervention delivery training

Half-day face-to-face training sessions were delivered to dental teams. Training sessions incorporated didactic learning, group discussions and role play to provide a basic understanding of the principles of behaviour change conversations and the RETURN intervention. Training was designed to be accessible to all by incorporating training methods to suit different learning styles.

### Phase 2: ‘on-the-job’ shadowing training

As all dental nurses do not routinely engage in undertaking behaviour change conversations (or even general discussions with patients), phase 1 training was supplemented by 1:1 individualised shadowing training. A key feature of this training was the coaching style, which fostered a supportive environment to build trainee confidence. The objective of this training was to attend to the individual training needs of different nurses and to ensure that all nurses were trained to the same level.

### Booster training and monitoring

An on-going training approach was adopted using booster training and reflective practice sessions. The need for booster training was continuously monitored through audio-recordings of the intervention sessions.

### Training the trainers

To maintain consistency in training provision, the RETURN training team (*n* = 4) attended a 3-day course with a clinical psychologist about how to deliver training on behaviour change conversations to dental teams. To maintain training standards throughout the training period, reflective practice sessions were conducted routinely amongst the RETURN training team. RETURN trainers also met regularly to discuss intervention delivery competency sign-off of dental team members, sharing scoring sheets and audio-recordings of intervention deliveries for discussion.

## Methods

### Ethics

Ethical approval was obtained from London-Camberwell St Giles Research Ethics Committee and the Health Research Authority (REC Ref: 21/LO/0059, IRAS ID: 288,546, 10/03/2021).

### Study design

This fidelity study used a mixed-methods design [30], integrating quantitative data from the RETURN training programme and qualitative data from observations, interviews with dental teams and intervention audio-recordings. Data were collected concurrently, analysed independently and then integrated to provide a comprehensive intervention fidelity assessment. A mixed-methods design was selected to enable a nuanced exploration of the factors influencing intervention fidelity, offering insights that would not have been attainable through a single-method approach [31]. Good Reporting of Mixed Methods Studies (GRAMMS) guidelines [32] were used to ensure comprehensive reporting of methods.

Given the limited existing knowledge on intervention fidelity within the dental care setting, a purposive sampling strategy was employed to maximise data capture and ensure a comprehensive analysis. All dental nurse interventionists were invited to participate in interviews, observations and training assessments and all available recordings of intervention delivery sessions were included in the analysis.

### Consent

Patients were consented as part of the RETURN trial study procedures [10]. The RETURN written informed consent procedure included a specific consent point where patients indicated that they were happy to have their intervention delivery session audio-recorded and used within this fidelity study.

Written informed consent was also obtained from the dental staff prior to the commencement of training by the lead author. The consent procedure was conducted face-to-face through the provision of a patient information sheet and consent form, and dental staff were afforded the opportunity to ask questions. Dental staff could withdraw from the study at any time, and this was reiterated throughout the study as part of the embedded ongoing consent processes.

### The fidelity assessment approach

The RETURN intervention was tailored, with trial participants effectively selecting which materials were included within their intervention session. In accordance with behaviour change conversation principles [33], participants were encouraged to lead sessions. Accordingly, trial participants determined how much or how little conversation took place. The intervention was designed so that no two sessions would be identical, although common guiding principles were used. As participants took the intervention pack home, all participants were afforded the opportunity to view all intervention materials.

### Training fidelity assessment methods

Training fidelity data were collected using a combination of training session attendance records, assessment forms (completed by both trainers and trainees) and trial activity logs. Additionally, the number of booster training and reflective practice sessions delivered to dental teams was documented. The specific training assessment measures used are outlined below:

- Training content checklists (Additional file 2) were used to determine whether the phase 1 training was delivered to dental teams as planned. Two RETURN trainers led each session: one delivered the training and the other acted as a secondary facilitator. Training content checklists were completed by the secondary facilitator to record whether essential training components were delivered (binary scores).
- Trainee evaluation forms (Additional file 3) were completed by individual trainees immediately after the phase 1 training. Evaluation forms asked trainees how satisfied they were with the training provision and were completed anonymously. Trainees evaluated the different training components, awarding scores between 0 and 5 for each. The form also provided opportunity for qualitative feedback. Scores were combined and reported as percentages.
- Trainee skill acquisition assessment forms (Additional file 4) were used by RETURN trainers to evaluate the skills demonstrated by dental teams during the phase 1 training. The criteria were based on training objectives, and RETURN trainers scored trainees’ skill acquisition on a scale from 0 to 3. Scores were combined and reported as percentages.
- RETURN Intervention Fidelity Checklists (Additional file 5) were used by RETURN trainers in two ways:
- During shadowing training to determine whether a trainee could be signed-off as competent to deliver the intervention independently (this occurred when a dental nurse achieved a score of 80% + in every check-list domain within a single delivery).
- Assessment of ‘skills drift’ by applying checklists to intervention audio-recordings and comparing dental nurses’ scores across three time points (immediately after competency sign off, in the middle of recruitment and their final intervention delivery). Scores were converted to percentages, with an overall score of 80% set as the threshold.

RETURN Intervention Fidelity Checklists employed a 4-point scale to indicate the degree to which an intervention component was delivered. A scale was used rather than binary scoring (i.e. present/not present) to provide an indication of competency across components. Criteria listed within the checklists were intervention specific and were derived from both the theoretical components of the intervention (i.e. MI spirit) and practical requirements of intervention delivery.

Guidelines were created to help the RETURN trainers to apply the checklist consistently.

### Semi-structured interviews and observations

Qualitative methods were used to capture the views and experiences of dental team members. Reporting of the qualitative work is guided by the consolidated criteria for reporting qualitative research (COREQ) checklist [34].

To capture the ‘real-time’ training experiences of the dental teams, informal, unstructured observations [35] were conducted by the RETURN training team throughout the training period (August 2021–April 2022). These were recorded in field notes at the end of each day. A standardised field note proforma and observation training was used. Regular team discussions were held, and an iterative approach was taken to facilitate flexibility as patterns emerged. Observation findings fed into subsequent semi-structured interview topic-guide design.

Semi-structured interviews (*n* = 13) were conducted by the lead author with the dental nurse interventionists at the end of the trial recruitment period, and interviews were centred around their experiences of delivering the trial and intervention. All interviews were conducted face-to-face in a private location within the dental nurses’ site (i.e. in a spare surgery or an empty staff room). Each nurse provided only one interview. Interviews were audio-recorded and transcribed. Audio files were deleted immediately after transcription. Interviews lasted 23–110 min depending on how much the participant wished to say. Participant identities were pseudo-anonymised in both the interview transcripts and the field notes.

Data were analysed using reflexive thematic analysis [36]. Analysis involved a process of coding the data using an inductive, data-driven approach to explore patterns of meaning, searching for similarities and differences. The coding was then organised into themes, with constant comparisons made with the whole data set. The data and analysis were managed using Microsoft Excel (version 2501, Microsoft Corporation). Due to clinical demands on the dental nurses, it was not possible to enact member checking. However, regular discussions were held between VL (not dentally trained) and JH (an experienced dental nurse) about the themes generated.

Reflexivity was essential, as data collectors had a pre-existing trainer-trainee relationship with the interventionists. To mitigate this power imbalance, the observation aims were disclosed to interventionists, and field notes were documented away from the setting. In the interviews, the interviewer attempted to shift power dynamics by verbally distinguishing their trial delivery and training experiences from the aims of the fidelity study, positioning all interviewees as ‘experts’ to be learned from.

### Delivery fidelity assessment methods

Dental nurses were provided with digital audio-recorders and asked to record all intervention sessions. Delivery fidelity was assessed by applying the RETURN Intervention Fidelity Checklist to the audio-recordings. Guidelines were created to standardise scoring, and the same two researchers were responsible for scoring throughout (VL and JH). As there were multiple (*n* = 14) interventionists, and the previously conducted RETURN feasibility study had found interventionist effects [24], all available audio-recordings were scored.

### Interrater reliability

Initially, 10 audio-recordings were randomly selected and double-scored. Scores were compared using the kappa statistic which yielded a value of 7.2, indicating substantial interrater agreement [37]. Thereafter, independently, 50% of the remaining recordings were scored each.

### Delivery fidelity assessment: primary and secondary outcomes

There are no guiding principles to inform how to categorise ‘high intervention fidelity’ in dental interventions. Accordingly, a cautious approach was taken. For an intervention to be considered as having been delivered with high fidelity, a fidelity threshold of 80% + within every intervention domain in a single session was set. This binary outcome (pass/fail) was labelled ‘fidelity threshold’ and was treated as the primary outcome in the delivery fidelity assessment.

It is acknowledged, however, that this is a high threshold for this type of intervention [38], and accordingly a secondary outcome was explored whereby intervention sessions achieving a score of 80% + overall (labelled ‘overall fidelity score’ and also treated as a binary outcome) were examined. The amount of intervention or ‘intervention dose’ was also calculated from the audio-recordings.

### Statistical analyses

Frequencies and percentages were used to summarise how many intervention delivery sessions achieved high levels of fidelity in accordance with the thresholds set by the primary and secondary outcomes. Fidelity within intervention criteria domains was explored to highlight fidelity variations and these were summarised using mean scores and standard deviations (SD).

To explore the association between the independent variables of interventionist, dose and barrier on fidelity outcomes, multiple logistic regression models were performed. These fixed effects in the model were selected based upon the RETURN feasibility study which suggested that those variables were likely to have substantive impacts on fidelity. In all the models, the nurse delivering the intervention was added as a random effect. For the primary outcome, the dependent variable was fidelity threshold (score of 80% + in every domain), coded as 1 where the threshold was achieved and 0 where it was not. For the secondary outcome, the dependent variable was overall fidelity score (score of 80% + overall), binary coded as 1 where the threshold was achieved and 0 where it was not. Confounders significantly associated with either of the fidelity outcomes at *p* < 0.05 level were included in the final regression models. Finally, a between-subjects’ analysis of variance (ANOVA) was conducted to examine any differences in dose between the six barriers, as a barrier effect was anticipated. These statistical analyses were conducted in R v. 4.3.1.

### Methodological ethical considerations

The participants of the fidelity study were the interventionists; however, the RETURN team were mindful of the ethical considerations presented through combining data streams in a mixed-methods design, and care was taken to maintain the confidentiality and anonymity of both the trial participants and the interventionists. This was especially important during the reflective practice component of the training, the interviews with the dental teams and during the audio-recording scoring.

## Results

The training fidelity assessment results are presented first, followed by the delivery fidelity assessment results.

### Training assessment results

Thirty-three dental staff took part in the phase 1 training and 28 dental nurses/previous dental nurses took part in the phase 2 training.

#### Standardisation of training

The same four trainers delivered all phase 1 training sessions, delivering 12, 3-h training sessions in accordance with the BCC guidelines. Training content checklists confirmed that every training session covered all necessary training components, and all trainees received the required training materials.

#### Dental nurse skill acquisition

The characteristics of the dental nurse trainees (such as gender, age, number of years dental experience) are detailed in Table 1.

**Table 1:** Characteristics, training information and interview status of the phase 2 RETURN trainees

Nurse code	Site	Gender	Age	Nursing experience (years)	Competency sign off?	Shadowing training hours received	Reason sign-off not achieved	Provided interview
01	RETURN Nurse	F	31	1	Yes	3	–	Yes
02	RETURN Nurse	F	33	10	Yes	3	–	Yes
03	RETURN Nurse	F	54	30	Yes	3	–	Yes
04	01	F	38	13	No	2.5	No time due to workload	Yes
05	01	F	42	21	Yes	7.75	–	Yes
06	01	F	31	17	Yes	9	–	Yes
07	01	M	61	17	Yes	9.5	–	Yes
08	01	F	26	6	Yes	5.15	–	No, on maternity leave
09	01	F	42	18	Yes	8.67	–	Yes
10	02	F	20	4	No	0	Left practice before shadowing commenced	No, left practice, not contactable
11	02	F	42	25	No	3	No time due to staff shortages	No, declined to take part
12	03	F	23	2	Yes	14.5	–	No, unable to contact
13	03	F	36	12	Yes	17.91	–	No, unable to contact
14	05	F	34	12	Yes	9.25	–	Yes
15	06	M	34	4	Yes	5.25	–	Yes
16	06	F	29	12	No	2.5	Maternity leave	No, on maternity leave
17	07	F	40	12	Yes	9	–	No, left practice, not contactable
18	07	F	62	Retired dental nurse	No	1	Nurse discontinued citing low confidence in own abilities	No, declined to take part
19	07	F	59	Retired dental nurse	No	1	Nurse discontinued citing low confidence in own abilities	No, declined to take part
20	07	F	68	Retired dental nurse	No	1	Nurse discontinued citing low confidence in own abilities	No, declined to take part
21	08	F	33	15	Yes	11.5	–	Yes
22	08	F	19	Trainee	No	8.5	Nurse discontinued citing low confidence in own abilities	No, declined to take part
23	08	F	19	Trainee	No	6.5	Nurse discontinued citing low confidence in own abilities	No, left practice, not contactable
24	08	F	21	2	No	1.25	Nurse discontinued citing low confidence in own abilities	No, left practice not contactable
25	09	F	31	5	No	2.75	No time due to staff shortages	No, declined to take part
26	11	F	20	1	No	1.75	Site discontinued NHS contract	No, lost contact due to discontinuation of NHS contract
27	12	F	24	4	No	7	Nurse discontinued citing low confidence in own abilities	Yes
28	13	F	46	27	No	6.25	No time due to staff shortages	Yes

Phase 1 trainees were mainly female (*n* = 31/33), had a mean age of 41 years (SD 16.2) and an average of 16.6 (SD 10.23) years dental experience. Phase 1 training assessment forms showed that only 50% of the site teams met the skills acquisition threshold. However, nearly all (98%, 32 of 33) reported that the training fully explained their project role, and 100% scored the training 5 out of 5 for meeting its learning objectives.

Phase 2 ‘shadowing’ trainees were mainly female (*n* = 26/28), had a mean age of 36 (SD 13.73) and had an average of 10.8 (SD 8.65) years dental experience. Fifty-two [14] percent of the trainees achieved competency sign-off. The total hours spent shadowing training was 152.5, and of those, 42.5 h was spent training nurses who did not complete the training. The number of shadowing training hours provided to individuals varied, with a mean of 9.77 h (SD 3.57), ranging between 5.15 and 17.91 h.

Table 1 shows that the two nurses who received the most shadowing training (nurses 12 and 13) had contrasting levels of experience (12 years and 2 years), and both were from site 03. This site also had the highest trial participant withdrawal rate due to baseline procedural issues (for example, unsigned consent forms, incorrect randomisation procedures), with 9 participants out of 58 (15%) withdrawn by the study team. These trial delivery challenges may have impacted on the amount of intervention training required for those nurses, as support for both was offered during the shadowing training. In contrast, the two nurses requiring the least shadowing training (nurses 08 and 15) represented relatively limited dental nursing experience (4 and 6 years) and were from different sites.

Low confidence was the primary reason for training non-completion, cited by 7 nurses (25%). Notably, all were either in training (*n* = 2), newly qualified (*n* = 2) or no longer practising as nurses (*n* = 3). Most were from sites 07 and 08 (*n* = 6), where trainees performed poorly in phase 1 training, particularly in behaviour change conversations and communication skills (scoring 0s and 1s in these domains). However, one nurse from each site (nurses 17 and 21) independently delivered the intervention multiple times, indicating that individual nursing experience, rather than site-specific skills gaps or external factors such as patient cohorts, was a key determinant of success.

In summary, this suggests that BCI delivery may be better suited to experienced, qualified and currently practising dental nurses. This also shows that the RETURN training programme was insufficient to bridge skills gaps associated with limited dental nursing experience (centred around communication skills), particularly in building confidence.

#### Minimisation of skills drift over time

Booster training and reflective practice sessions were used to prevent skills drift. Five booster training sessions and 16 reflective practice sessions were delivered throughout the trial. Each nurse attended at least one reflective practice session.

**Fig. 1:**
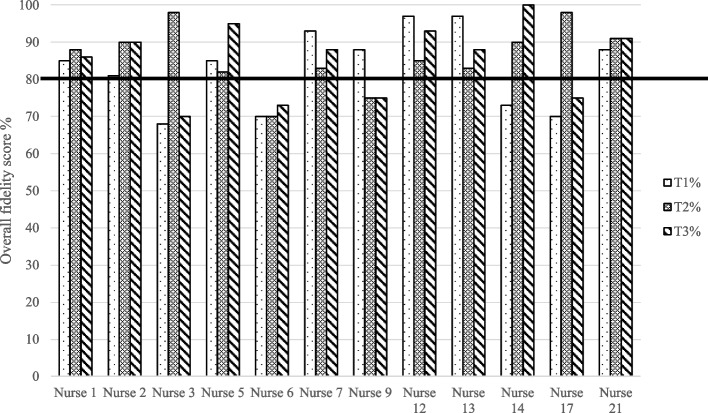
Dental nurse fidelity score percentages over time

Skills drift was low. Figure 1 shows overall fidelity score percentages for the dental nurses at three time points (T1 = immediately after achieving sign off, T2 = mid-way through recruitment and T3 = their last intervention). The bold black line marks the 80% fidelity score threshold. Scores for nurses 08 and 15 were not included due to missing intervention delivery audio-recordings.

One nurse scored below 80% at all time points and four scored below 80% at least once. However, all nurses who scored below the threshold improved over time. Nine nurses achieved higher scores in T3 compared to T1 indicating that the RETURN training approach effectively prevented skills drift.

#### Qualitative results: experiences of the RETURN training

Thirteen dental nurses were interviewed, and 58 training sessions were observed. Despite attempting to interview all the dental nurses involved in the delivery of the RETURN intervention, this was not possible. Table 1 outlines the reasons for missing interviews, most commonly due to staff turnover.

Three themes were identified: (1) research naivety increased overall training needs, (2) confidence was the key to success, and shadowing training supported this and (3) wider trial IT requirements were a barrier to intervention training.

#### Theme (1): research naivety increased overall training needs

None of the dental nurses had been involved in delivering a research project prior to RETURN (including the nurses employed as float recruiters), and many felt this inexperience slowed training progress.

I remember not quite understanding what we were doing [during the intervention delivery training], but I think that’s because I’d no research background in the beginning. I think that’s why it took a while for things to click for me. DN 09, Site 01

I learn more being hands on, practical, so I think when [the trainer] was coming in, I was picking it up, but quite slow. I’d not done anything like that before, I didn’t even know these things existed, so it was an eye opener. DN 27, Site 12

This finding suggests that it would be beneficial to take prior research experience into account when designing training for future research trials involving dental nurse interventionists.

#### Theme (2): confidence was the key to success, and shadowing training supported this

Most of the dental nurses felt the shadowing training was where they were able to build their confidence to deliver the intervention competently.

I thought that [shadowing training] was good and actually essential because it was, totally out of your comfort zone because you’ve never done it before….you know…. opening up that conversation with the patient. It was good to have that back up, and you know, spend the time to build the confidence. If you had of set me loose after the afternoon we spent [intervention delivery training], I think I might have froze in front of the patient. DN 28, Site 13

One dental nurse also explained this helped with their confidence concerning safeguarding risks when delivering the intervention.

I was worried about what to do if something were to go wrong, like what if there was a risk of suicide, or like a history of abuse, what would I do. But having that person there to guide me through the first few, that was invaluable, that helped my confidence. I learned how to navigate the conversation. DN 15, Site 06

This finding shows the value the dental nurses placed on the support offered through the shadowing training provision. One aim of the shadowing training component was to address differences in confidence between the dental nurses, and this finding suggests this objective was met for some nurses.

#### Theme (3): wider trial IT requirements were a barrier to intervention training

The third theme identified was that most of the dental nurses found using the IT systems for trial processes difficult. One nurse indicated that the IT got in the way of their intervention delivery training:

It was a lot if you’re only doing it once a week like I was. I felt like I was going back to square one each time, trying to remember which log on, or what to click next. I’m not that IT minded really. It really was a faff, and I think worrying about the IT did get in the way of me making progress with the conversation with the patient. DN 07, Site 01

One senior dental nurse provided some insights into IT use within dental nursing roles, which could suggest this should be limited in the implementation of future dental trials:

So, I think it’s because, it’s my day-to-day job to kind of access the kind of [IT] systems and you know do all the kind of IT side of things, I’m kind of used to that, whereas obviously some of our nurses, particularly our Band 4 nurses, it’s just not within their remit. DN 04, Site 01

### Delivery assessment results

A total of 1179 participants were recruited to the RETURN trial, and 591 participants were allocated to receive the RETURN intervention. Of those, 7% [39] elected not to receive the full intervention (most commonly due to wishing to leave the clinic or because they were in pain) and instead received a ‘short delivery’ where the patient pack was provided, but no discussion, video or action planning activities were undertaken. A total of 78% (462) of the interventions were audio-recorded and subsequently fidelity scored. Missing recordings were due to the intervention delivery taking place during the training period [40], the dental nurse forgetting to record the session [15], technological issues such as audio-recorder malfunctions or corrupted audio files [21] or where no reason was provided [35].

#### Dental visiting barrier

The most common barrier selected by the trial participants was ‘I don’t think to go when I’m not in pain’ (38%, 188), followed by ‘anxiety’ (26%, 130). The remaining barriers made up relatively smaller proportions: ‘cost’ (9%, 44), ‘time’ (8%, 39), ‘embarrassment’ (6%, 29) and ‘trust’ (5%, 26).

#### Dose

Dental nurses were encouraged to keep to a 15-min intervention delivery. Mean intervention dose was 15.80 min (SD = 6.72; range 0–47 min).

#### Comparing intervention dose between barriers

Due to the personalised nature of the intervention, dose variation was anticipated. To explore this, a between subjects ANOVA was performed which revealed that there was a significant difference in time spent delivering the intervention across the different barriers (*F*(5,450) = 9.88, *p* < 0.001, *η*^2^ = 0.10). A boxplot visualising the differences both within and between the intervention barriers can be found in Additional file 6. Overall, this shows that the barrier of ‘embarrassment’ had the most variation in dose and the barrier of ‘time’ had the smallest variation and was also associated with the shortest intervention deliveries. In addition, the barriers of ‘anxiety’, ‘cost’, ‘embarrassment’ and trust’ were all similar in dose.

Next, specific dose differences were explored through Holm-corrected *p* values, which can also be found in Additional file 6. This analysis shows that there were multiple barriers where intervention dose was significantly different from another barrier. For example, those receiving the ‘time’ intervention were more likely to have received less intervention than those receiving an ‘embarrassment’, ‘anxiety’ or ‘cost’ intervention.

#### Delivery fidelity outcomes

For the primary outcome of fidelity threshold (those intervention deliveries that achieved a score of 80% + in every domain), 12.1% [41] of the interventions met this threshold, and 87.9% (406) did not. For the secondary outcome of overall fidelity score (those intervention deliveries that achieved an overall score of 80% +), 79.9% (369) of the intervention deliveries met this threshold, and 20.1% (93) did not.

Table 2 shows overall mean fidelity scores broken down by intervention domain. Two of the domains (overarching communication skills and setting goals and actions plans) reached mean scores of over 80%, with a third (increasing motivations) sitting marginally below this threshold. The three remaining domains achieved scores of around 75% or more.

**Table 2:** Mean fidelity scores for the different RETURN intervention domain

RETURN intervention criteria domain	Mean %	Standard deviation
Overarching communication skills	84.48%	26.67%
Discussion about dental attendance barriers	75.78%	29.34%
Increasing motivation through the video	79.65%	30.35%
Increasing knowledge via barrier booklet	77.02%	27.23%
Setting SMART goals and action plans	82.35%	27.82%
Increasing intentions	74.97%	19.21%

#### Interventionist, barrier and dose

For the following logistic regression analyses, ‘short deliveries’ were excluded to mitigate multicollinearity. As the data were nested (participants within nurses delivering the intervention), a variance components model was performed to test the necessity of adding the nurse as a random effect. The model with the random intercept (AIC = 451.44) was significantly better than the null model (no random intercept; AIC = 460.58, χ^2^(1) = 11.14, *p* < 0.001). Therefore, the subsequent analysis included nurse as a random effect.

A model with barrier as a predictor was performed. This model (AIC = 440.42) was significantly better than the random intercept only null model (AIC = 451.44; χ^2^(5) = 21.02, *p* < 0.001, correctly classifying 80% of cases). Next, intervention dose was added as a predictor to the model. This model (AIC = 389.66) was better than the model with barriers only (AIC = 440.42; χ^2^(1) = 52.76, *p* < 0.001 correctly classifying 82% of cases). When intervention dose was added, several barrier effects were no longer significant, with the barrier of ‘cost’ remaining significant and dose also acting as a significant predictor for this final model, shown in Table 3.

**Table 3:** Regression analysis for the primary outcome (fidelity score of 80% + in each domain)

Predictors	Odds ratios	95% CI	*p* value
Cost	0.25	0.08–0.80	0.019
Don’t think to go	0.85	0.46–1.56	0.595
Embarrassment	1.19	0.45–3.18	0.729
Time	0.49	0.14–1.80	0.283
Trust	0.83	0.28–2.46	0.741
Dose (min)	1.23	1.16–1.30	< 0.001
Marginal ***R***^2^ 0.294			

#### The relationships of delivery nurse, barrier and dose to overall fidelity score

Again, this was nested (participants within nurses delivering the intervention), so a variance components model was performed. The model with the random intercept (AIC = 416.91) was significantly better than the null model (no random intercept; AIC = 446.48, χ^2^(1) = 31.57, *p* < 0.001). Therefore, subsequent analysis included nurse as a random effect. A model with barrier as a predictor was performed. This model (AIC = 417.85) was not better than the random intercept only model (AIC = 416.91; χ^2^(5) = 9.06, *p* = 0.107), with the model with barrier correctly classifying 23% of cases. This time, none of the barriers was significant predictors. Thereafter, intervention dose was added as a predictor to the model. This model (AIC = 383.46) was better than the model with barriers only (AIC = 417.85; χ^2^(1) = 36.38, *p* < 0.001 correctly classifying 27% of cases). Dose had a strong association with overall fidelity score, as shown in Table 4.

**Table 4:** Regression analysis for the secondary outcome (fidelity score of 80% + overall)

Predictors	Odds ratios	95% CI	*p* value
Cost	0.91	0.32–2.59	0.857
Don’t think to go	0.96	0.48–1.91	0.911
Embarrassment	5.08	0.58–44.63	0.143
Time	0.91	0.35–2.40	0.856
Trust	0.53	0.16–1.78	0.307
Dose (min)	1.25	1.15–1.36	< 0.001
Marginal *R*^2^ 0.270			

## Discussion

This article presents the training and delivery intervention fidelity assessment of a BCI delivered by dental nurses within a pRCT. To the authors’ knowledge, this is the first comprehensive intervention fidelity assessment of a trial testing a BCI within the dental setting. First, the three main assessment findings are discussed, followed by a discussion around the study’s strengths and limitations, and finally future study directions and recommendations are summarised.

### Dental nurses delivered the RETURN intervention with high levels of fidelity

Although few interventions reached the primary fidelity outcome threshold score of 80% + in every domain (12.1%), most interventions achieved the less stringent secondary fidelity outcome of scoring 80% + overall (79.9%). Additionally, the intervention criteria domain-level fidelity exploration found that all domains were delivered with mean fidelity scores of around 75% or higher. Taken together, although the pre-defined primary fidelity threshold was often not met, this study provides evidence that the RETURN intervention was delivered with high fidelity.

Intervention dose variations were apparent, which is not an unusual finding in highly tailored interventions [26, 31, 32]. We found the more intervention delivered by nurses, the higher the chances of reaching the fidelity threshold, but that dose variations were associated with the selected barrier. Moreover, we found the barrier of ‘cost’ negatively impacted fidelity scores. If we put this into the context of the RETURN intervention (i.e. patient-led, MI-style), insights emerge for how highly tailored behaviour change interventions should be assessed for fidelity. For example, the barrier of ‘cost’ could be considered a structural rather than behavioural barrier (such as anxiety or embarrassment), and accordingly, ‘cost’ did not ‘fit’ well with the theoretically derived (MI) fidelity scoring framework. It is possible that nurses found it difficult to offer supportive statements about next steps when a participant indicated they did not attend the dentist due to financial barriers (as opposed to offering support for dental anxiety, say). This suggests for multi-component/multi-topic interventions, flexible fidelity scoring mechanisms that allow for variations within the same intervention would be better.

Additionally, considering the patient-led ethos of the intervention (and the accompanying fidelity scoring) perhaps patient factors affected fidelity scores within the barrier of ‘cost’. For example, previous research found that patients categorised as low socio-economic status (SES) (around 50% of the RETURN participants) are less likely than their high SES counterparts to take the lead in medical consultations [42]. In RETURN, fidelity scores were awarded where attributes such as ‘non-directive talk’ were present and for facilitating participants to come up with their *own* barriers and setting their *own* goals and action plans. It could be that those trial participants most likely to select the ‘cost’ barrier were also more likely to be from low SES backgrounds, and therefore the nurses (as inexperienced MI/behaviour change interventionists) may have found it difficult to facilitate a participant-led session, thereby dropping fidelity scores. This suggests that careful consideration should be given to trial populations when designing and interpreting training and fidelity scoring systems.

Whilst there are very few intervention fidelity assessments in the dental literature, a study delivered in family centres in rural locations in the USA evaluated the fidelity of a MI intervention aimed at controlling early childhood caries. This study reported variability in MI skill application and dosage amongst counsellors, aligning with our findings [43]. They noted that counsellors achieved a competency rating around the midpoint of their scale, whilst the dental nurses in our trial demonstrated higher fidelity ratings of between 75 and 85%. However, direct comparisons between the two studies are challenging due to differences in fidelity measures and conceptualisation of competency within our assessment framework.

In the broader health literature, behavioural interventions delivered by healthcare staff in primary care settings yield comparable findings to our study. For instance, a nurse-led primary palliative care oncology study reported an average fidelity rating of 75% [44]. Similarly, a behaviour change intervention aimed at increasing physical activity amongst patients at risk of cardiovascular disease achieved an overall fidelity rating of 85.4% for intervention components and 75% for behaviour change techniques [39]. Additionally, a walking intervention implemented by practice nurses and healthcare assistants in primary care demonstrated that 78% of the intervention components were delivered as intended [45]. Two of those studies [39, 45], however, achieved comparable fidelity ratings with less resource intensive training approaches than was required in RETURN, which indicates that different types of interventions and different settings may require different levels of effort to achieve acceptable levels of intervention fidelity.

### Dental nurses with previous patient-facing experience were best suited to delivering the intervention

Results indicate that the RETURN training was delivered in accordance with the BCC recommendations [13, 14]. Training, however, was not without its challenges. Just 50% of the dental site teams achieved the 80% pass rate for the phase 1 (standardised) intervention training, and only 52% of the dental nurses completed the phase 2 (individualised) training. The main reason for phase 2 training drop-out was because the dental nurses lacked confidence in their ability to deliver the intervention. Those who elected to discontinue training due to confidence issues were either trainees, relatively newly qualified (under 5 years) or not currently practising dental nurses.

As only half of the dental teams met the skill acquisition threshold for the standardised training, this demonstrates the importance of a tailored training approach for dental nurses, a point which is supported by our qualitative findings. Indeed, tailored training approaches have been found to be successful in confidence building in nurse interventionists previously [39, 46], especially where intervention training consolidates or develops existing skills [46]. Effective communication skills were a big part of successfully delivering the RETURN intervention with high fidelity, and during the RETURN feasibility study, baseline variations in these skills were found [24]. A tailored training approach was therefore implemented for this study to ‘plug the skills gap’ for the dental nurses.

We conclude, however, that the RETURN training programme was not sufficient to develop missing skills, and nurses who achieved competency sign off to deliver the intervention independently likely already had a good baseline level of communication skills. By comparison, a trial testing a similar intervention to RETURN successfully trained dental nurses to deliver a MI intervention in secondary care. However, that study found differential outcomes by site which were acknowledged, but not investigated in-depth [47]. Considering the type of intervention delivered in RETURN, we focused on the dental nurse as the key unit of analysis, rather than site. This approach highlighted several factors crucial for future implementation of dental nurses as interventionists, particularly that not all dental nurses are equally suited to meet research expectations. Other multi-site RCTs delivering BCIs in dental settings, however, have only incorporated site effects into their statistical modelling [22, 48, 49] or have not reported site or interventionist effect explorations within their analyses at all [50].

In summary, nurses with regular patient interaction are best suited as BCI interventionists, and early site discussions should ensure their selection to optimise training efficiency and intervention success.

### Disentangling trial delivery burden from fidelity assessment

Interventions delivered in pRCT designs cannot be procedurally separated from the requirements of the trial. Indeed, the design of pRCTs delivered by healthcare workers in healthcare settings are a balancing act, where trial process burden must be balanced with intervention delivery effort [51]. One novel finding from this study’s qualitative work was that routine IT use was not part of the usual day-to-day role requirements for the RETURN dental nurses. The nurses felt that the IT requirements of the trial tasks impacted upon both intervention training length (and therefore the amount of resource allocated to training) and training success (as confidence was impacted by poor IT skills). Therefore, it is possible that intervention training success could have been improved if trial processes were better aligned with the skills of the dental team, or if responsibility for trial processes and intervention delivery were separated, e.g. by employing research nurses to deliver trial tasks.

A previous study which conducted both a patient-level RCT and independently, a cluster RCT in primary dental care settings, testing the same dentist-delivered behaviour change intervention, yielded statistically significant clinical results in the cluster RCT only [52]. The authors surmise that one potential reason was that dentists in the cluster RCT design were better at delivering the intervention as they were more practised. It could also be the case that there was less ‘trial burden’ in the cluster RCT, with no randomisation element ‘muddying the training waters’, and more training effort could be focused on intervention delivery. Given that dental practices are relatively ‘research naïve’ settings [53], complying with the trial protocol is an additional threat to intervention fidelity.

Whilst there are general discussions in the literature about the burden of clinical trials on healthcare systems and healthcare staff, broadly concluding that pRCTs require specialist planning to ensure that procedures align with the contextual environment [54, 55], there is a dearth of studies looking at the specific issue of RCT burden and its effects on intervention fidelity. It is logical that this may be more problematic in areas where there is less research activity (and therefore less prior research experience), such as dental practices. We accordingly recommend that further work is needed in intervention fidelity research in the dental setting to address the impact that trial procedural burden can have on intervention fidelity. It would be useful to consider these concepts separately, perhaps through the employment of process evaluations focusing on trial implementation.

## Strengths and limitations

### Potential disadvantages of a comprehensive approach to fidelity assessment

An important consideration within the context of this study, and its broader implications for the RETURN trial outcomes, is the possible repercussions of implementing a comprehensive approach to intervention fidelity enhancement, monitoring and assessment on trial results. For instance, did the extensive assessment and monitoring methods influence the behaviours of interventionists, and would we see different effectiveness results if we had simply delivered the training and assessed the resultant outcomes? This issue has received limited attention in the fidelity literature to date [53]. However, Nelson and colleagues provide an insightful discussion on this topic in their paper outlining a procedure for assessing intervention fidelity in behaviour change experiments. They argue that when researchers actively manipulate intervention fidelity, the concept must be reinterpreted to acknowledge that the observed outcomes may not be replicable in ‘real-world’ settings without researcher support [56].

The decision to implement an in-depth intervention fidelity strategy within the RETURN trial was guided by the research team’s prior experience in conducting trials within the challenging, research naïve, primary dental care settings [22]. It also aimed to respond to consistent findings in systematic reviews of oral health behaviour change interventions that highlight a lack of methodological rigour, particularly around intervention procedures which hinder the ability to draw conclusions about which interventions are effective, and which are not [19, 41, 57]. An ‘on balance’ approach was taken, and it was determined that the additional information to be garnered through an extensive intervention fidelity assessment would be invaluable in terms of informing what would be needed for real-world implementation (for example, this study has revealed the most suitable characteristics for dental nurse interventionists delivering BCIs). Some mitigating actions were taken to create a sense of ‘distance’ between the research team, the interventionists and the fidelity assessment, however, such as the use of interventionist-administered audio-recordings, rather than in-person observational methods.

### Strengths

A key strength of this study was the large amount of fidelity data captured through intervention audio-recordings—a gold-standard data capture method [13]. However, as not all sessions were recorded, it is possible that the delivery fidelity assessment was not fully representative, and it is also possible that dental nurses only recorded/retained the recordings of their ‘best’ intervention deliveries as they were aware the recordings would be scored. Some confidence can be taken, however, from the large number of available recordings, representing 78% of the interventions delivered. A further strength is the comprehensiveness of the fidelity assessment approach in accordance with a fidelity framework and the use of a variety of methods and measures within the assessment. The use of mixed methods enabled a deeper understanding of intervention fidelity and provided tangible, nuanced insights which informed the recommendations made.

### Limitations

Some limitations of the study should be noted. The work focuses on an intervention delivered by dental nurses working in England, within the NHS dental system. The role of dental nurses and dental system contexts may differ in other countries, so this may have implications for generalisability. For example, in the USA, the role of dental assistants varies by state according to regulatory frameworks in place, with some accredited as Expanded Function Dental Assistants who have expanded independent clinical work delivery responsibilities [58].

Due to resource constraints, it was not possible to employ independent fidelity scorers and intervention trainers, which is acknowledged as the gold-standard approach [13]. In terms of delivery fidelity, it is possible that it was difficult for the fidelity scoring team (some of whom were also involved in the intervention design, study implementation and intervention delivery and training) to maintain impartiality, and that this may have introduced bias into the fidelity scoring. For example, it is possible that those who worked on the training and implementation of the study may have had a vested interest in its ‘success’, and therefore, inadvertently scored sessions through a positive lens.

However, precautionary measures such as reflexivity and positionality considerations were embedded and implemented to address this concern, alongside other measures such as interrater reliability and reflective practice amongst the scoring team. In addition, fidelity assessments were conducted after the trial follow-up was finalised. This was an additional measure designed to minimise potential bias, as RETURN researchers were also involved in the trial’s unblinded follow-up calls. It could also be argued, however, that members of the research team were well-placed to undertake the fidelity assessment, as they had an intimate knowledge of the intervention’s objectives, theoretical grounding and practical requirements. Indeed, it is common within the design of fidelity assessments to employ the use of the research team or those who delivered the intervention as fidelity scorers [40, 59, 60].

This fidelity assessment also included intervention deliveries by the ‘float’ dental nurse recruiters, who were employed as part of the research team. It is possible that these nurses delivered the intervention with greater fidelity than practice-based nurses, who had to integrate research activities alongside their routine clinical responsibilities. The ‘float’ dental nurses may have been more familiar with the intervention protocol and had fewer competing demands on their time ensuring a singular focus on the delivery of the research. Consequently, they may have been able to dedicate more time and attention to ensuring high-quality intervention delivery, potentially influencing fidelity outcomes. On balance, however, the practical decision to employ ‘float’ dental nurses was made in response to the challenges posed by the COVID-19 pandemic at the time of the study commencement (August 2021), particularly issues related to staff availability within dental settings. To mitigate potential biases introduced by their role in intervention delivery, the float nurses were specifically recruited directly from dental practices, ensuring that none had prior research experience. This approach aimed to maintain relevance to real-world practice in terms of their representative skills, whilst addressing the practical constraints of RCT implementation during the pandemic.

It was not possible to interview all the dental nurse interventionists involved in the RETURN trial. The most common reason for this was due to staff turnover. Staff retention of dental nurses within NHS dental practices is often problematic, with reasons such as poor career progression opportunities cited [61]. As this study did not include all the dental nurse interventionists, it is feasible that those who partook were more positive about their experience of the delivery of the trial (hence they were willing to take part). The qualitative data generated from this work stream, however, did include a mix of views, both positive and negative, and so a range of viewpoints were captured.

Finally, the fidelity checklist used to score the interventions did not undergo validity and reliability testing and its accuracy and consistency cannot be guaranteed. Whilst there are scales available for measuring MI within interventions [62], the intervention itself was not designed as a purist MI intervention, and other theoretical frameworks were also integrated [9], making scoring complex. The fidelity checklist created was accordingly, intervention specific. To mitigate this, however, attempts were made to generalise the criteria contained within the scoring checklist so that many of the areas scored would be applicable to other BCIs, particularly those implementing MI spirit.

### Summary of implications and future research directions

The authors propose several recommendations for researchers implementing complex interventions in RCTs within real-world, primary dental care settings:

1. For complex interventions involving multiple components or tailored content, fidelity assessment tools should incorporate adaptable scoring criteria to account for variations in delivery whilste maintaining methodological rigour.
2. When designing fidelity strategies and assessments, researchers should carefully consider the characteristics of the target patient population receiving the intervention (e.g., SES, education level, health literacy). These factors may influence intervention fidelity, and should therefore, be factored into fidelity study designs.
3. Different interventions and settings may necessitate varying levels of training to achieve acceptable fidelity. Early planning of fidelity strategies and assessment is essential, incorporating site involvement from the outset. Discussions on site suitability should include key interventionist-related factors, such as prior experience in patient-facing roles for more complex BCIs.
4. In trials where intervention delivery relies on the skills of interventionists, both interventionist effects and site-specific factors should be explored.
5. Further research is needed to explore the impact of trial-related procedural demands on intervention fidelity within dental practice settings. Strategies to minimise procedural burden should be considered to enhance fidelity and feasibility.
6. Further work is required to embed research skills and training within primary dental care settings more generally.

These recommendations aim to inform the design and evaluation of future complex interventions in primary dental care, improving the reliability and applicability of findings.

## Conclusion

To the authors’ knowledge, this is the first comprehensive intervention fidelity assessment of the training and delivery of a complex BCI delivered by dental nurses in primary care dental settings. This study has shown that it is possible to train dental nurses to deliver a complex brief BCI in the urgent dental setting with high levels of fidelity alongside their usual clinical roles. However, not all dental nurses are suited to this type of patient interaction. Consideration should be given to dental nurses’ previous patient-facing experience and communication skills. This will enhance the chances of training success, resulting in interventions that are delivered as they were intended. Careful consideration should also be given to the type of intervention and fidelity scoring framework use, paying particular attention to context and trial populations. Finally, research in this setting should focus on fidelity assessment in ways that are detached from the procedural burdens inherent in RCTs. This will provide information that will better inform intervention adoption into dental settings beyond research trials. The work undertaken within this study has made a significant contribution to the primary dental care literature by providing a comprehensive examination of intervention fidelity. The recommendations and insights derived from this work will enhance the methodological rigour of future trials conducted in this complex and challenging setting.

## Supplementary Information

Below is the link to the electronic supplementary material.

Additional file 1: RETURN training programme overview. Further information about the training provision for the RETURN trial

Additional file 2: Training content checklist. Checklist used to determine what training content was delivered by the RETURN trainers

Additional file 3: RETURN study intervention training evaluation. Form used by RETURN trainees to evaluate training received

Additional file 4: Intervention delivery training assessment checklist. Assessment form used by RETURN trainers to record skill acquisition demonstrated by trainees during phase 1 training

Additional file 5: RETURN fidelity checklist. Fidelity checklist used to assess intervention fidelity

Additional file 6: Boxplots and Holm-corrected *p* values. Boxplots and Holm-corrected *p* values showing additional information about intervention dose

Additional file 7.

## Data Availability

The qualitative data sets generated during this study are not publicly available due to confidentiality and anonymity considerations. The anonymised quantitative data set is available upon reasonable request from the authors.

## Abbreviations

pRCT: Pragmatic randomised controlled trial
RETURN: Interventions to reduce inequalities in the uptake of routine care
BCI: Behaviour change intervention
UK: United Kingdom
BCC: National Institutes of Health Behaviour Change Consortium
MI: Motivational interviewing
REC: Research Ethics Committee
SD: Standard deviation
ANOVA: Analysis of variance
USA: United States of America
