# Supplementary figures and images for: A complex behaviour change intervention delivered by dental nurses: mixed-methods fidelity assessment of the RETURN intervention
Source: Trials. 2025 May 13;26:156. doi: 10.1186/s13063-025-08856-0 (PMC12070712; doi:10.1186/s13063-025-08856-0)

**Additional file 1: RETURN training programme overview**

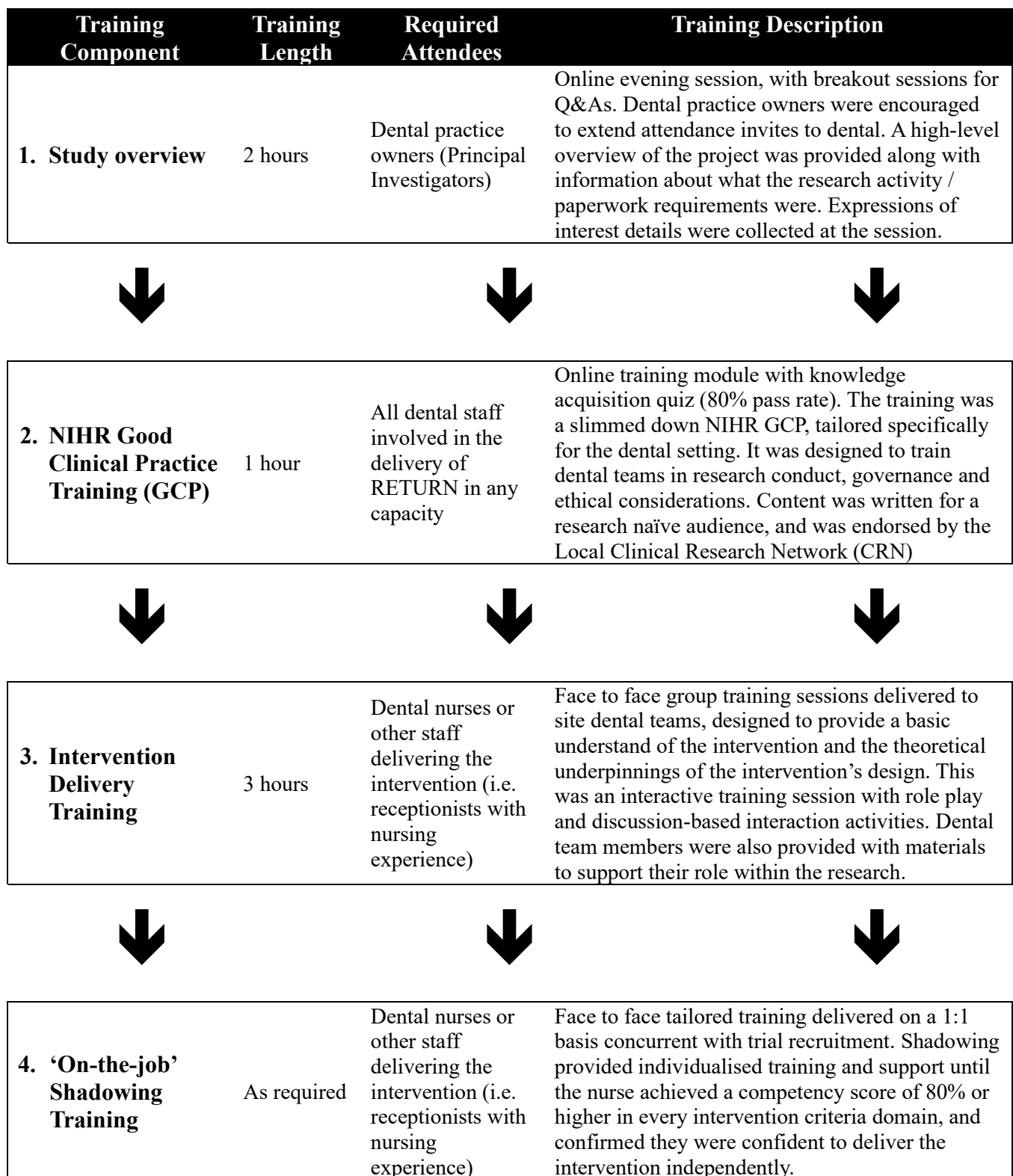

Supplement: Supplementary file 1 — Additional file 1: RETURN training programme overview. Further information about the training provision for the RETURN trial [file 13063_2025_8856_MOESM1_ESM.pdf]
