# Supplementary material for: A complex behaviour change intervention delivered by dental nurses: mixed-methods fidelity assessment of the RETURN intervention
Source: Trials. 2025 May 13;26:156. doi: 10.1186/s13063-025-08856-0 (PMC12070712; doi:10.1186/s13063-025-08856-0)
Supplement: Supplementary file 2 — Additional file 2: Training content checklist. Checklist used to determine what training content was delivered by the RETURN trainers [file 13063_2025_8856_MOESM2_ESM.pdf]

**Additional file 02**

**RETURN Training Content Checklist used by RETURN trainers to determine whether the training content was delivered as it was intended**

**Training session date:**

**Trainers:**

**Site:**

**Attendees (name and job role):**

| **Training Component** | **Delivered** | **Not delivered** |
|---|---|---|
| Standardised RETURN Intervention training slide desk used throughout the session | | |
| A description of the RETURN intervention and the dental nurse role | | |
| Overview of inequalities in dental attendance, and the health determinants that feed into this | | |
| Group task designed to encourage thought around the steps and difficulties involved in behaviour change | | |
| Overview of behaviour change theory with group discussion about personal experiences of this | | |
| Overview of behaviour change conversations in a healthcare setting, with group participation | | |
| Overview of effective communication skills with group discussions about personal experiences of this | | |
| Interaction with the RETURN intervention materials | | |
| Overview of SMART goals and action plans | | |
| Role play exercise of intervention deliveries using at least 2 case vignettes | | |
| Hard-copy training pack provided to all trainees | | |
| Hard-copy patient intervention pack provided to all trainees | | |
| Training website address details provided to all trainees | | |
