# Supplementary material for: A complex behaviour change intervention delivered by dental nurses: mixed-methods fidelity assessment of the RETURN intervention
Source: Trials. 2025 May 13;26:156. doi: 10.1186/s13063-025-08856-0 (PMC12070712; doi:10.1186/s13063-025-08856-0)
Supplement: Supplementary file 3 — Additional file 3: RETURN study intervention training evaluation. Form used by RETURN trainees to evaluate training received [file 13063_2025_8856_MOESM3_ESM.pdf]

Additional File 03

RETURN intervention training evaluation completed by trainees who attended the half-day intervention training session

RETURN study Intervention Training Evaluation

Aim of the session: To develop an understanding of the psychological intervention used in the RETURN Trial and competency in delivering this effectively within a dental setting

Learning outcomes:
1. To understand the overall aim of the RETURN trial and the role of the dental nurse
2. To understand the main barriers to routine dental visiting
3. To increase knowledge on what influences health behaviours and behaviour change
4. To develop skills in setting SMART goals and action plans
5. To develop skills in empathetic, non-judgemental communication with patients
6. To increase knowledge on research governance processes relevant to a randomised controlled trial
7. To develop skills in data collection processes involved in the RETURN trial

	Please rate training accordingly	Lowest → highest					
		0	1	2	3	4	5
1	The training met my expectations						
2	The training met the learning outcomes						
3	The topics covered were relevant						
4	Time allocated for training was sufficient						
5	Overall rating of the quality of presentation material and training manual						
6	Did you find group discussion (vignettes) useful?						
7	Did you find the training fully explained your role in the RETURN trial?						
8	What did you like best?						

9	Was there anything that you did not like or think we can improve?
10	Any other comments?

A verifiable CPD certificate will be sent to you
