# Supplementary material for: A complex behaviour change intervention delivered by dental nurses: mixed-methods fidelity assessment of the RETURN intervention
Source: Trials. 2025 May 13;26:156. doi: 10.1186/s13063-025-08856-0 (PMC12070712; doi:10.1186/s13063-025-08856-0)
Supplement: Supplementary file 4 — Additional file 4: Intervention delivery training assessment checklist. Assessment form used by RETURN trainers to record skill acquisition demonstrated by trainees during phase 1 training [file 13063_2025_8856_MOESM4_ESM.pdf]

Additional File 4

Trainee Skill Acquisition Assessment Forms used by the RETURN trainers to evaluate the skills demonstrated by the dental nurse trainees during the phase 2 training

Site Name:	**Score**			
**Training Component**	0	1	2	3
Demonstrated a basic understanding of factors that feed into health determinants				
Demonstrated a basic understanding of behaviour change conversations				
Demonstrated a basic understanding of how to convey emotional understanding of a patients' feelings and context				
Demonstrated a basic understanding of what good communication skills markers are				
Demonstrated a basic understanding of each of the RETURN intervention components				
Demonstrated intervention skills listed above through role play using case vignettes				
