# Supplementary material for: A complex behaviour change intervention delivered by dental nurses: mixed-methods fidelity assessment of the RETURN intervention
Source: Trials. 2025 May 13;26:156. doi: 10.1186/s13063-025-08856-0 (PMC12070712; doi:10.1186/s13063-025-08856-0)
Supplement: Supplementary file 5 — Additional file 5: RETURN fidelity checklist. Fidelity checklist used to assess intervention fidelity [file 13063_2025_8856_MOESM5_ESM.pdf]

Additional file 5

RETURN fidelity checklist

**RETURN fidelity checklist**<br><br>**Dental Nurse Code:**	**Fully Implemented** Score: 3	**Substantially Implemented** Score: 2	**Partially Implemented** Score: 1	**Not implemented** Score: 0
**Overarching communication skills**				
Use of empathic listening statements				
Use of relevant open questions				
Use of non-judgemental language				
Use of non-directive talk				
Patient's priorities, beliefs and challenges acknowledged				
**1: Discuss barriers to regular dental attendance – raise awareness**				
Patient given space to tell their story (if they choose to do so)				
Patient encouraged to come up with their own barriers without being led, or spoken for				
Patient encouraged to decide on the one barrier they want to work on				
**2: Increase Motivation**				
Patient is shown the video relevant to their selected barrier				
Encouragement provided to the patient to reflect on the video, and how their own situation relates				
**3: Increase knowledge**				
Patient guided to the booklet relevant to their selected barrier				
Information (relevant to the barrier) provided to the patient				
Statements communicated offering hope and assurances to the patients about their ability to overcome barriers				
Emphasis placed on the benefits of regular dental attendance				
**4: Setting SMART goals and action plans**				
SMART principles applied to goal and action plan				
Patient guided to set their own goal and action plan tailored to their situation				
Photographs of goal and action plan taken				
**5: Increase Intention**				
Encouragement statements about what's been achieved in the session				

Encouragement provided to the patient to look at intervention materials at home					
**Feedback:**					
**Strengths**					
**Areas for development**	Unhelpful components present in session:<br>☐ Providing directive clinical advice<br>☐ Telling the patient what they should or should do / have done<br>☐ Setting goal / action plan for the patient<br>☐ Choosing the barrier for the patient				
