# Supplementary material for: A complex behaviour change intervention delivered by dental nurses: mixed-methods fidelity assessment of the RETURN intervention
Source: Trials. 2025 May 13;26:156. doi: 10.1186/s13063-025-08856-0 (PMC12070712; doi:10.1186/s13063-025-08856-0)
Supplement: Supplementary file 6 — Additional file 6: Boxplots and Holm-corrected p values. Boxplots and Holm-corrected p values showing additional information about intervention dose [file 13063_2025_8856_MOESM6_ESM.pdf]

Additional file 6

Boxplots showing the relationship between dose and barrier

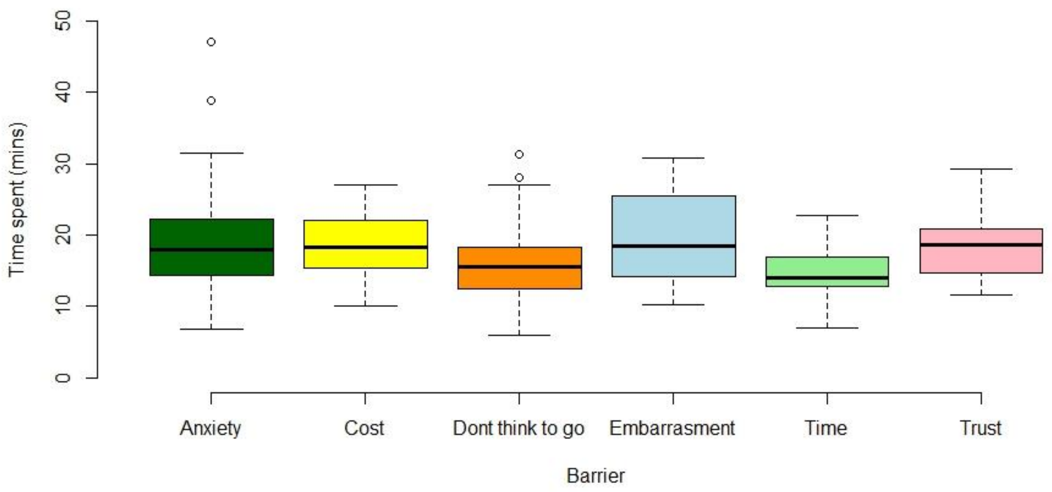

Dose differences using Holm-corrected p-values

Barrier comparisons	Estimate	SE	Df	t.ratio	p.value
Anxiety – Cost	0.4758	0.866	450	0.549	1.000
**Anxiety – Don't think to go**	**2.8131**	**0.567**	**450**	**4.965**	**<.0001**
Anxiety – Embarrassment	-1.4112	1.020	450	-1.383	0.788
**Anxiety – Time**	**4.2079**	**0.907**	**450**	**4.640**	**0.0001**
Anxiety – Trust	0.5420	1.067	450	0.508	1.0000
**Cost – Don't think to go**	**2.3374**	**0.832**	**450**	**2.810**	**0.0466**
**Cost – Time**	**3.7321**	**1.092**	**450**	**3.416**	**0.0076**
**Don't think to go – Embarrassment**	**-4.2243**	**0.991**	**450**	**-4.263**	**0.0003**
Don't think to go – Time	1.3948	0.874	450	1.596	0.7788
Don't think to go – Trust	-2.2711	1.039	450	-2.185	0.2352
**Embarrassment – Time**	**5.6191**	**1.218**	**450**	**4.613**	**0.0001**
Embarrassment – Trust	1.9532	1.342	450	1.456	0.7788
**Time – Trust**	**-3.6659**	**1.258**	**450**	**-2.915**	**0.0374**

95% CI
